# Cross-Sectional Study Protocol for the COVID-19 Impact Survey of Mothers and Their 7–11 Year Old Children in Alberta, Canada

**DOI:** 10.3389/fpsyt.2021.597759

**Published:** 2021-06-22

**Authors:** Nicole Letourneau, Sheila McDonald, Lyndsay Jerusha MacKay, Rhonda C. Bell, Erin Hetherington, Andrea J. Deane, Deborah Dewey, Sarah Edwards, Catherine J. Field, Gerald F. Giesbrecht, Susan Graham, Catherine Lebel, Brenda Leung, Sheri Madigan, Brae Anne McArthur, Carly McMorris, Nicole Racine, Kharah M. Ross, Muci Wu, Suzanne C. Tough

**Affiliations:** ^1^Faculty of Nursing, University of Calgary, Calgary, AB, Canada; ^2^Department of Paediatrics, Cumming School of Medicine, University of Calgary, Calgary, AB, Canada; ^3^Department of Community Health Sciences, Cumming School of Medicine, University of Calgary, Calgary, AB, Canada; ^4^Owerko Centre at the Alberta Children's Hospital Research Institute, University of Calgary, Calgary, AB, Canada; ^5^Department of Psychiatry, Cumming School of Medicine, University of Calgary, Calgary, AB, Canada; ^6^Faculty of Agricultural, Life and Environmental Science, University of Alberta, Edmonton, AB, Canada; ^7^Department of Radiology, Cumming School of Medicine, University of Calgary, Calgary, AB, Canada; ^8^Department of Psychology, Faculty of Arts, University of Calgary, Calgary, AB, Canada; ^9^Public Health Program, Faculty Health Sciences, University of Lethbridge, Lethbridge, AB, Canada; ^10^Werklund School of Education, University of Calgary, Calgary, AB, Canada; ^11^Psychology, Centre for Social Sciences, Athabasca University, Athabasca, AB, Canada

**Keywords:** COVID-19, mental health, well-being, child, family, risk, resilience (psychological), maternal

## Abstract

**Objectives:** Our aim is to understand the effect of the COVID-19 pandemic on families who have been followed longitudinally in two cohorts studied in Alberta, Canada. We will examine household infections during the COVID-19 pandemic, financial impact, domestic violence, substance use, child school and daily life and relationships in the home. We will identify risk and protective factors for maternal mental health outcomes using longitudinal data that can inform policy and government resource allocation in future disasters.

**Methods:** Mothers who are currently participating in two longitudinal studies, Alberta Pregnancy Outcomes and Nutrition (APrON; *N* = 1,800) and All Our Families (AOF: *N* = 2,534) were eligible to participate. Mothers were invited to complete the baseline COVID-19 Impact Survey (20–30 min) within 4 months of March 15, 2020, which was when the province of Alberta, Canada, implemented school closures and physical-distancing measures to prevent the spread of COVID-19. Mothers were asked to report on their own, their child's and their family's functioning. Mothers were re-surveyed at 6 months after completion of the initial COVID-19 Impact Survey, and will be re-surveyed again at 12 months.

**Results:** Responses from participants in both cohorts will be examined in harmonized analyses as well as separately. Descriptive, multivariable analysis will be undertaken to examine risk and resiliency over time and factors that predict mental health and well-being.

**Conclusions:** This study will provide timely information on the impact of COVID-19 for Albertan families. It will identify risk and protective factors for mental health and well-being among contemporary urban families supported by a publicly funded health care system to inform allocation of resources to support those most vulnerable during a global pandemic.

## Introduction

The COVID-19 pandemic has placed families under significant stress due to disrupted economic instability, changes to family routine and fear of infection. The lives of families living in Alberta Canada have been dramatically impacted by the COVID-19 pandemic and may never return to normal. Some families may experience increased detrimental impacts from the COVID-19 pandemic due to the following risk factors: food insecurity, domestic violence, alcohol and substance use, children's home schooling, parental responsibilities, children's social interactions, children's screen time, COVID-19 household infections, financial concerns and maternal distress. However, some families may not be as deeply impacted by the COVID-19 pandemic due to the following protective factors: activity of daily living, eligibility for provincial/national assistance, childcare, resiliency and coping and maternal relationship satisfaction. As Albertans aim to survive and recover from the impacts of the COVID-19 pandemic, understanding how the risk and protective factors have impacted outcomes is needed.

On March 15, 2020, physical distancing orders were put in place by the Alberta Provincial government in Canada to prevent the spread of COVID-19. Early estimates suggest increased levels of anxiety, depression and stress during the COVID-19 pandemic due to fears of contamination, infection, grief, stress, boredom and consequences of social and economic chaos (1–6). It is recognized that many people living in the province of Alberta, Canada are experiencing increased mental health difficulties during the current COVID-19 pandemic, such as anxiety, stress and depression, along with problematic family relations and lifestyle challenges (7, 8). This places children at risk for poor outcomes, especially vulnerable children living in at-risk environments with the increased instability from the COVID-19 pandemic (9–13). A cross-sectional study conducted among families (*N* = 361) living in Ontario, Canada found that during the COVID-19 pandemic children engaged in increased screen time and decreased physical activity, families adopted unhealthy behaviors such as eating more and parents reported moderately high levels of stress from financial instability and balancing work and childcare/homeschool (14). Parents indicated that they were concerned about their children's increased difficult behaviors and that they found it extremely difficult to balance homeschooling, working full-time hours from home and their parenting role (14). In a study conducted in Quebec, Canada among 144 families, 77% of parents reported changes in employment status, 44% reported changes in family income and 69% reported changes in sleep habits (15). Increased parent fear about COVID-19 was significantly related to child fear about COVID-19, and families who experienced changes in family income experienced increased concern about financial resources (15). Brown et al. found that parents (*N* = 183) in the US experienced increased anxiety and depressive symptoms during the COVID-19 pandemic and that increased parental anxiety and depressive symptoms were associated with increased child abuse potential (16). Importantly, parents who experience increased anxiety and depressive symptoms are more likely to exercise harsher disciplinary practices (13). Families are experiencing multiple negative impacts during the COVID-19 pandemic. This study will use utilize longitudinal data from two large existing cohort studies to understand and demonstrate the impact of the COVID-19 pandemic on mental health and well-being of families living in Alberta.

As a result of the COVID-19 pandemic, family life has been drastically altered because schools are closed, children are learning from home, group activities are ceased and parents are working remotely, unable to work or have become unemployed. Such alterations to family life are contributing to increased parental stress and disruptions in family relations (17–20). Parents are dealing with the demands of homeschooling their children while meeting employment requirements (18), which has increased their caregiver burden, increased their anxiety and resulted in lower closeness and greater conflict with their children (21). Prolonged school closure and isolation at home has resulted in negative effects on the physical and mental health of children (4, 14, 16, 19, 21). Children's behaviors with Attention Deficit Hyperactivity Disorder (ADHD) have significantly worsened during the COVID-19 pandemic compared to their normal state (22). A large cross-sectional study of COVID-19's impact on families with children aged two to 12 living in Hong Kong (*N* = 29,202) found that as a result of school closures children are spending extended time on electronic devices, experiencing increased psychosocial problems, decreased prosocial behavior and poor functioning (23). Also, parents exhibited increased level of parenting stress and children were delayed in going to bed, engaged in decreased recreational activities and physical exercise (23). Increased parental stressors amidst decreased supports has placed children at risk of increased abuse and domestic violence (16, 17, 24, 25) and placed women in abusive relationships at even greater risk (24). One study in the United Kingdom found an increase in children treated for suspected abusive head trauma (26). Another study found that parents who lost their jobs, experienced depression and previously psychologically maltreated their children were more likely to psychological maltreat their children during the COVID-19 pandemic (27). The COVID-19 pandemic has placed families under unprecedented stressors and they have experienced significant alterations to family relations, which has resulted in negative impacts for children (e.g., abuse, harsh discipline, physical and mental health difficulties and disruptive behaviors). Thus, there is a pressing need to document the effects that the COVID-19 pandemic has on families, children and adolescents in Alberta.

This COVID-19 study draws upon two on-going pregnancy cohort studies in Alberta: the Alberta Pregnancy Outcomes and Nutrition (APrON) cohort (*N* = 2,189) and the All Our Families (AOF) cohort (*N* = 3,387). The APrON study is an ongoing longitudinal cohort study of pregnant mothers, their partners and children in Alberta, Canada, that recruited mothers from 2009 to 2012 (28, 29). This cohort study focuses on identifying the influences and intersections between maternal mental health (depression, anxiety, stress) and nutrient status during pregnancy and postpartum on: (1) birth and obstetric outcomes (e.g., prematurity and low birth weight), (2) infants' and children's health and development (e.g., neuropsychological and mental health) and (3) maternal mental health over the longer term. Mothers were surveyed during pregnancy in each trimester. Mother-child pairs were followed up when children were 3, 6, 12, 24 and 36 months, 5 and 8 years of age, with proposed follow-up at 12 and 16 years of age. Data are currently being collected at the 8-year assessment. For APrON methods see Kaplan, Giesbrecht (29) and for preliminary results see Leung, Giesbrecht (28). For the 90+ published articles on the APrON cohort go to https://apronstudy.ca/journal-articles/.

The AOF study is a community-based, ongoing prospective pregnancy cohort focused on examining maternal well-being and mental health, infant outcomes and risks and protective factors for child development and psychosocial health (30). AOF recruited participants from 2008 to 2011. Data have been collected regarding labor and deliver, birth outcomes, breastfeeding, maternal physical and mental health, health care utilization, family well-being, community resource use, child health and development, lifestyle, parenting and the child care environment (30). Mothers were surveyed during pregnancy at <25 weeks and 34–36 weeks. Mother-child pairs were followed up when children were 4 months, 1, 2, 3, 5 and 8 years of age. Data have been collected for the 8-year follow up. Mothers and youth will be contacted again when the children are 13 years of age. For AOF methods see Tough et al. (30) and preliminary results see McDonald et al. (31). For 80+ articles published on the AOF cohort go to http://allourfamiliesstudy.com/publications/.

**Figure 1:**
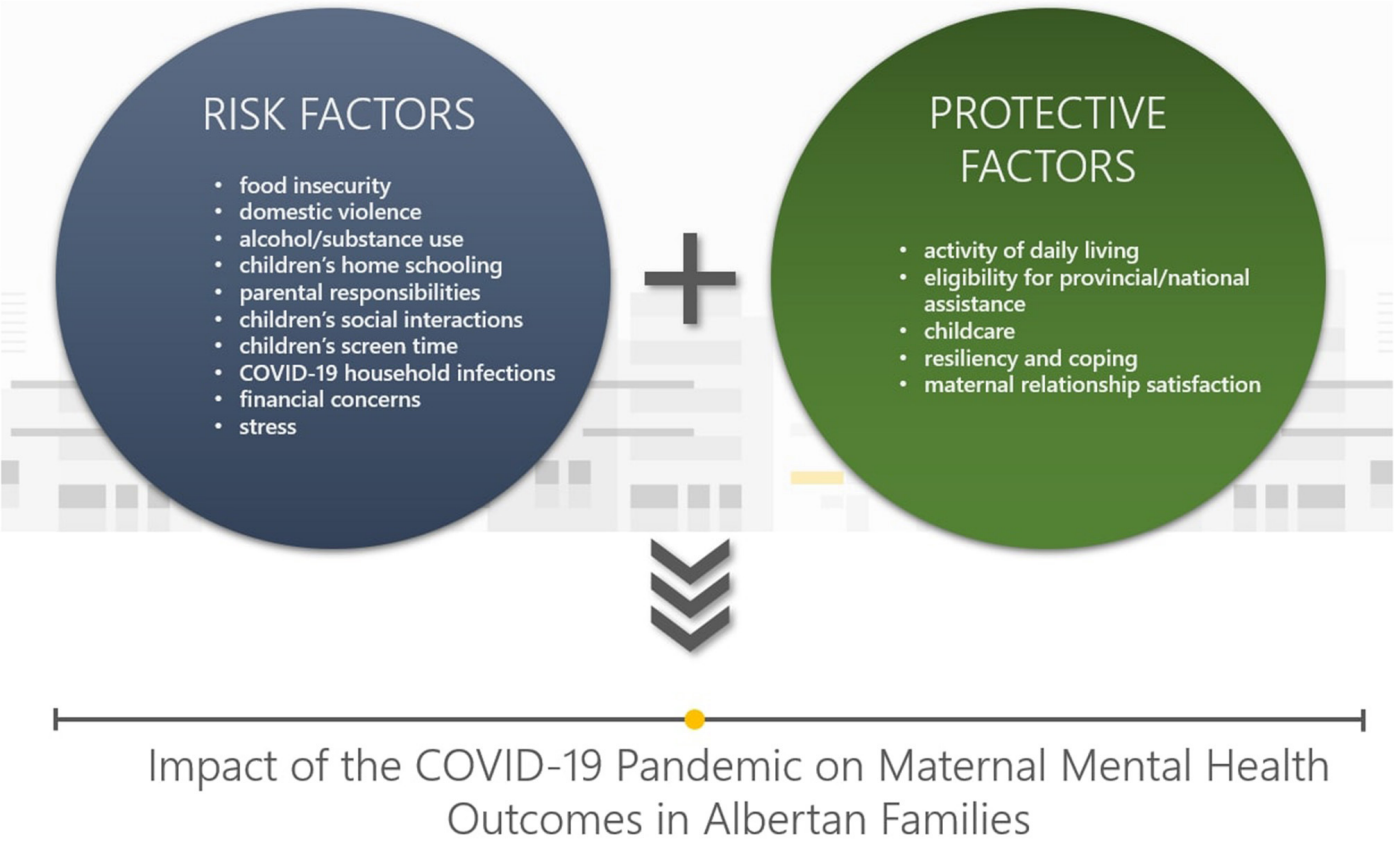
Risk and protective factors that influence impacts of the COVID-19 pandemic on maternal mental health in Albertan families.

Given overlap in content areas in APrON and AOF, the cohorts harmonized data collection for the 5- and 8-year questionnaires. This provides an opportunity to investigate the effect of COVID-19 on maternal mental health in families participating in these studies. We are uniquely positioned to explore emerging risk and protective factors during the pandemic as we will leverage pre-pandemic longitudinal data to compare maternal mental health outcomes before and during COVID-19 (see Figure 1). Specifically, the study will:

1. Describe household infection during COVID-19;
2. Describe family experiences of the pandemic within 3–4, 6, and 12 months of the outbreak across the areas of financial impact, domestic violence, substance use, maternal mental health and well-being, child school and daily life, relationships in the home and demographics;
3. Determine the risk (i.e., food insecurity, domestic violence, alcohol and substance use, children's school instruction in the home, parental responsibilities, children's social interactions, children's screen time, COVID-19 household infections, financial concerns, stress) and protective factors (i.e., activity of daily living, eligibility for provincial/national assistance, childcare, resiliency and coping, maternal relationship satisfaction) associated with maternal mental health outcomes in families over the course of the COVID-19 pandemic;
4. Provide contemporary evidence to inform policy and programs to support health and development of families living in Alberta during the COVID-19 pandemic.

## Methods

### Design

This is a surveillance study of Albertan families with children age 7–10 years in the APrON cohort and 8–11 in the AOF cohort as of March 2020, when the public health policies regarding physical distancing and school closures related to the COVID-19 pandemic were instated in Alberta, Canada. The baseline COVID-19 Impact Survey was sent to APrON participants on May 26, 2020 and AOF participants on May 20, 2020. The baseline COVID-19 Impact Survey questionnaire was completed by July 31 for APrON and July 15 for AOF, that is, within 3–4 months of March 15, 2020, which was when Alberta implemented strict physical-distancing measures to prevent the spread of COVID-19. Repeat cross-sectional COVID-19 Impact Surveys have been sent out at 6 months after the Medical Officer of Health implemented population based physical distancing orders, and another COVID-19 Impact Survey is scheduled to be sent out at 12 months after implementation of physical distancing orders. Funding has been attained to aggregate data for cross sectional analysis at the first wave of data collection. APrON and AOF will launch a harmonized follow up questionnaire, the results of which could be examined in harmonized analyses as well as separately. Ethics approval to conduct this additional survey, at the multiple time points, was obtained for both studies. Potential unintended consequences include adding to the burden of families already stressed by COVID-19 and increasing the risk of attrition in the overall APrON and AOF studies.

This study is a unique collaboration across two longitudinal cohorts who agreed upon the development of a standardized questionnaire. This questionnaire was circulated to content experts, subspecialists and other cohort leaders who reviewed it to increase the likelihood that core data elements could be harmonized across other studies at this time.

The baseline COVID-19 Impact Survey was programmed into Research Electronic Data Capture (REDCap) and pilot tested. REDCap is a secure data center within the Faculty of Medicine and Dentistry at the University of Alberta. It is a web-based electronic data capture solution with servers located under Canadian jurisdiction. Researcher access to the survey data is a combination of role-based access, strict password management processes and two factor authentication. Programming of the survey into REDCap was time sensitive and reflected broad collaboration across disciplines and expertise. Collection of identical data across cohorts will enable more robust data analysis and increase the validity of the findings.

### Eligibility

Participants were eligible to participate if they were currently enrolled in the APrON or AOF cohorts and had consented to be contacted for future research. Initial eligibility for the APrON cohort included pregnant women at least 16 years old, <27 weeks gestation at time of recruitment, living in or near Calgary or Edmonton and able to complete written questionnaires in English (29). Initial eligibility for the AOF cohort included women <25 weeks gestation at time of recruitment, at least 18 years old, were accessing prenatal care in Calgary, Alberta and were able to complete written questionnaires in English (30).

### Expected Sample Size

COVID-19 Impact Surveys were sent to 1,800 APrON participants and 2,534 AOF participants in May 2020. Based on previous waves of our longitudinal data collection we expect at least a 70% response rate. Therefore, the expected sample size for this study is 3,033.

### Procedures

E-mail invitations were sent to eligible participants with a cover letter describing the study, an online consent form and a hyperlink to direct participants to REDCap to complete the questionnaire. Paper copies of the questionnaire were sent to eligible participants enrolled in the APrON study who have indicated a preference for traditional mail, rather than email for participation.

### Participant Reminders

Participants were sent email reminders to encourage questionnaire completion. Beginning 10 days after the initial baseline COVID-19 Impact Survey invitation e-mail, reminder e-mails were sent out to participants from the APrON study every week for 5 weeks, with July 26, 2020 the last day to complete the baseline questionnaire. Four weekly email reminders were sent to participants from the AOF study, and non-responders were contacted by telephone. The last day for AOF participants to complete the questionnaire was July 15, 2020.

### Remuneration for Participation

As per historical practices, participants from the APrON study were not offered incentives to participate in this study while participants from the AOF study were offered a $20 gift card.

### Consent

Participants were consented at baseline during the initial COVID-19 Impact Survey, via the online consent form, prior to completing the questionnaire. All invited participants were asked to consent to be contacted for future research.

### Data Management

Study data will be managed using REDCap and housed at the University of Alberta. Data from paper questionnaires will be entered into REDCap by APrON volunteers. Data from APrON and AOF cohorts will be downloaded from REDCap for cleaning and data analysis. Data from baseline questionnaires will be pooled into a single data set for descriptive and multivariable analysis. Qualitative short answer responses will be extracted, stored and organized in NVIVO. All data including that from follow-up questionnaires is managed separately by the two cohort teams according to existing research and ethics protocols.

### COVID-19 Impact Survey

Instrument and variable selection for the COVID-19 Impact Survey were predicated on previously used instruments and scales in order to facilitate direct comparison with previously collected data. Demographic variables included: number of people living in the household and total income before COVID-19 for all household members. Standardized tools were used when available. The baseline COVID-19 Impact Survey also included researcher derived questions that were developed with expert consensus and aligned with longitudinal data pertaining to risk factors (i.e., job loss, sector of employment, food insecurity, domestic violence, alcohol and substance use, educational instruction of children in the home, managing parental responsibilities, children's social interactions, children's screen time) and protective factors (intent to vaccinate, eligibility of provincial or national assistance, activities of daily living during COVID-19, childcare, relationships inside the home). Questions drawn from established or validated questionnaires are described below.

#### Risk Factors

##### Alberta Childhood COVID-19 Cohort Study

Items on household infections during COVID-19 were adapted from the AB3C study (32), which was developed by content experts in infectious disease and pediatrics.

*Household Infections During COVID-19:* Mothers were asked if they or household members were infected by COVID-19 (yes, no, maybe; confirmed by laboratory test, used an online or telephone assessment tool, had symptoms consistent with COVID-19 infection, had contact with someone with a confirmed test, other), characterization of household members' symptoms (mild, moderate, severe), whether a member of extended family or a close friend had COVID-19 (yes, no) and if someone close to respondent passed away after being infected by COVID-19 (yes, no).

##### Impacts of COVID-19 (CPSS-COVID)

Items were adapted from the Statistics Canada Perspective Survey Series 1, using the CPSS-COVID (33). Our team assessed it to have face and content validity relevant to our participants.

*Financial Impact:* Mothers were asked their perspectives of the impact of COVID-19 on their ability to meet their financial obligations/needs (major impact, moderate impact, minor impact, no impact, too soon to tell).

*Concerns:* Mothers were asked about their concerns regarding impacts of COVID-19 on members of the household, Canadians and the world population. Questions also addressed maternal concerns in the areas of civil disorder, social ties, ability to cooperate and support one another, family stress and violence in the home. Items were rated on a 4-point Likert scale from “not at all” to “extremely.”

##### Perceived Stress Scale

The PSS-10 was employed (34). This scale was used to assess maternal stress as it has strong reliability with a Cronbach's alpha of 0.89 and was found to have construct validity (35).

*Maternal Stress:* Mothers were asked how often they have been or felt: upset, unable to control things, nervous, confident coping with problems, able to control irritations in their life, on top of things, anger and as though difficulties were piling up. Items were rated on a 5-point Likert scale from “never” to “very often.”

#### Protective Factors

##### Brief Resilient Coping Scale

Items were employed from the (BRCS) (36). Cronbach alpha's has been found to be 0.76 and test-retest reliability 0.71 (37).

*Maternal Coping:* Mothers were asked if the following described their behavior or actions in the past 2 weeks: looked for creative ways to alter difficult situations, believed they were in control of their reactions, grew from difficult situations and actively looked for ways to replace losses. Items were rated on a 5-point scale from “does not describe me at all” to “describes me very well.”

##### Dyadic Adjustment Scale

A single item was used from the DAS (38), which has been found to be reliable with an overall scale Cronbach's alpha of 0.96 (39) and reported valid among adult heterosexual couples (38).

*Maternal Relationship Satisfaction:* Mothers were asked about their degree of happiness in their relationships with their spouse/partner on a 7-point scale from “extremely unhappy” to “perfectly happy.”

#### Other Assessments

Additional questions included in the survey sent to APrON participants assessed topics such as maternal pregnancy status, support from partner and maternal-reported child behavior/mental health.

Responses to open-ended questions will be collected about the following: factors that would impact mothers' decisions to vaccinate or not, experiences with assistance programs, comments about drinking alcohol, cannabis use and recreational drug use, things that are going well with maternal mental health and well-being, maternal support of children's school goals and well-being, maternal perspectives on children's connection with friends and family through social networking or online platforms and anything else about the mothers' family experiences during COVID-19, such as alternative arrangements, changes to their lifestyle or anything important about how they and their family are managing.

#### Maternal Mental Health Outcomes

##### Spielberg State-Trait Anxiety Inventory

Items were employed from the six-item short-form of the **state** scale of the STAI (40). The mean test-retest reliability has been determined at 0.89 (41). Validity was found among the graduate-level educational environment (42).

*Maternal Anxiety:* Mothers were asked if they currently felt: calm, tense, upset, relaxed, content and worried. Items were rated on a 4-point intensity scale from “not at all” to “very much so.”

##### Center for Epidemiologic Studies Depression Scale

We employed the CES-D-10 within the baseline COVID-19 Impact Survey (43). The CES-D-10 has been found valid among healthy community dwelling older adults (44), test-retest reliability has been found to be 0.41–0.70 and Cronbach's alpha's 0.80–0.86 (45).

*Maternal Depressive Symptoms:* Mothers were asked if they felt or behaved: bothered, unable to focus, depressed, activities took an effort, hopeful, fearful, restless, sleep, happy, lonely and motivated. Items were rated on a 4-point Likert scale from “rarely or none of the time” to “most or all of the time.”

## Analysis Approach

### Quantitative Analysis

Data will be exported from the REDCap platform for data cleaning (e.g., SPSS). Missing data will be examined and multiple imputation methods applied as appropriate. Data will be analyzed using SPSS Version 24.0 (46). Reliability and validity will be evaluated of items from the COVID-19 Impact Survey. Descriptive and multivariable analysis will be undertaken to examine risk and resiliency over time and factors that predict mental health and well-being. Multivariable analysis will be undertaken to explore impacts on co-morbid outcomes (e.g., depression and anxiety) (47); however, other analyses will explore impacts on unique mental health and well-being outcomes. Further, we will compare the families who respond to the COVID-19 Impact Survey to those that did not, to determine if there are significant socio-demographic differences that may impact findings.

### Qualitative Analysis

Responses from open-ended questions will be analyzed using thematic analysis to identify common themes. Thematic analysis is a qualitative data analysis method used for identifying, analyzing and reporting reoccurring patterns or themes within data (48).

## Dissemination of Outcomes

Results from the baseline COVID-19 Impact Survey will be reported immediately to inform decision-making and planning for families and children in Alberta and across Canada with respect to the original study aims. Organizations for dissemination include: Maternal Infant Child and Youth Research Network (MICYRN), Public Health Agency of Canada, Canadian Pediatric Society, Alberta Health Services Strategic Clinical Networks, Calgary Reads, First 2000 Days Network, United Way and Parent Link Centres. Newsletters containing a plain language summary of study findings will be created and posted on APrON and AOF websites. Results will be submitted for publication in peer reviewed journals and shared at academic conferences and via media outlets.

Results from the 6- and 12-month surveys will be provided to decision makers and stakeholders and inform future APrON and AOF research. These data will be examined within each cohort to examine longitudinal impacts of COVID-19 exposure on maternal mental health outcomes in families, with predictions based on unique cohort variables. If funding allows, data from each cohort will be aggregated longitudinally for analysis.

## Future Research and Data Sharing

Findings from the baseline COVID-19 Impact Survey analysis will be available to advance research among international cohort consortiums including: EU Lifecycle, Research Advancement through Cohort Cataloguing and Harmonization (ReACH) and Australian Research Alliance for Children and Youth (ARACY). Data will be shared with the Maternal Infant Child Youth Research Network (MICYRN) COVID-19 catalog and World Health Organization Maternal Newborn, Child and Adolescent Health COVID-19 Research Network. As we become aware of other international research teams collecting similar data we will be open to collaboration. Detailed data documentation are available upon request.

Current processes and protocols are in place for sharing and accessing study data as previously established by the APrON and AOF studies. Contact principle investigators (AOF: Tough, APrON: Letourneau) for more information.

## Recruitment Progress

This study was launched on the 20th of May, 2020. As of the 15th of July, 2020, 1756 eligible participants had completed the baseline COVID-19 Impact Survey.

## Ethics Statement

The studies involving human participants were reviewed and approved by University of Calgary Conjoing Health Research Ethics Board (Project numbers: AOF: REB13-0868_MOD14, APrON: REB14-1702_MOD25) and University of Alberta Health Research Ethics Board (Pro00002954). The patients/participants provided their written informed consent to participate in this study.

## Author Contributions

NL, ST, SMc, SMa, NR, SE, and EH contributed to the drafting of the study protocol. NL, ST, SMc, EH, AD, DD, CM, GG, KR, and MW contributed to the development of study materials, including the baseline COVID-19 Impact Survey. NL, LM, ST, and SMc wrote and revised sections of the manuscript. All authors are part of the ongoing APrON and AOF study and COVID-19 Impact Survey teams, reviewed, offered editorial suggestions and approved the final version of the manuscript.

## Conflict of Interest

The authors declare that the research was conducted in the absence of any commercial or financial relationships that could be construed as a potential conflict of interest.
